# Storage Potential of the Predatory Ladybird *Cheilomenes propinqua* in Relation to Temperature, Humidity, and Factitious Food

**DOI:** 10.3390/insects13070613

**Published:** 2022-07-07

**Authors:** Sergey Ya. Reznik, Andrey N. Ovchinnikov, Olga S. Bezman-Moseyko, Konstantin G. Samartsev, Natalia A. Belyakova

**Affiliations:** 1 Zoological Institute, Russian Academy of Sciences, Universitetskaya 1, 199034 St. Petersburg, Russia; anovchi@gmail.com (A.N.O.); bezman-moseyko@mail.ru (O.S.B.-M.); ksamartsev@gmail.com (K.G.S.); 2 All-Russia Institute of Plant Protection, Russian Academy of Sciences, Podbelskogo 3, Pushkin, 196608 St. Petersburg, Russia; belyakovana@yandex.ru

**Keywords:** biological control, storage, survival, fecundity, hatching, temperature, humidity, starvation, *Cheilomenes propinqua*

## Abstract

**Simple Summary:**

Short- and long-term storage potential is an important feature of a biocontrol agent. We estimated survival during storage in the absence of natural food (aphids) and the post-storage fecundity of females of the predatory ladybird *Cheilomenes propinqua* in relation to temperature (from 7 to 27 °C), air humidity (from 50 to 80–90%), and feeding regime (starved or fed). The experiments showed that under the optimal conditions (temperatures of 15–17 °C and feeding on the grain moth eggs) *C. propinqua* females can be stored for up to 80 days for future use in mass rearing or the biological control of pests. Short-term storage or transportation (shipment) can occur at a much wider range of temperatures, i.e., from 12 to 27 °C. We conclude that *C. propinqua* can be successfully used for biological control in greenhouses by preventing colonization, although this would require the regular supplementation of food. Besides this, storage for more than 10 days at temperatures of 7 °C and lower results in 100% mortality; therefore, escaped individuals would not survive the winter even in the south of the temperate zone.

**Abstract:**

The ability of the females of the predatory ladybird *Cheilomenes propinqua* to survive and to retain reproductive potential in the absence of natural food (aphids) was estimated under various hydrothermal (temperatures of 7, 12, 17, 22, and 27 °C; air humidities of 50% and 80–90%) and trophic (starved vs. fed on the frozen eggs of the grain moth *Sitotroga cerealella*) conditions. The post-storage reproductive potential was estimated using the mean number of eggs laid over 20 days. The experiments showed that fed females can be stored at temperatures of 15–17 °C for 20 days with a rather low (about 20%) and for up to 80 days with an acceptable (not more than 50%) loss of the reproductive potential. The successful short-term (up to 3 days) storage or transportation of fed and starved females can occur at temperatures from 12 to 27 °C. However, storage for more than 10 days at temperatures of 7 °C and lower results in the 100% mortality of both the starved and fed beetles. These data suggest that (1) in greenhouses, *C. propinqua* can be used for the biological control of aphids by preventing colonization, although this would require the regular supplementation of factitious food, and (2) escaped individuals would not survive the winter even in the south of the temperate zone.

## 1. Introduction

The augmentative biological control of greenhouse insect pests, which is the most promising alternative to the extensive use of chemical pesticides, involves the mass rearing of a biocontrol agent [1,2,3,4,5]. The ability to survive in the absence of natural food is a substantial advantage of a mass-produced insect. This ability is important in two rather different processes: storage and transportation (shipment). The need for the storage of a biocontrol agent arises in a number of situations: a low-demand period, a need to accumulate insects for a large delivery, or a temporary lack of natural food, etc. [6]. In all of these cases, storage is usually a long-term process (up to several months) taking place in a mass-rearing facility under more- or less-controlled favorable conditions. Transportation (delivery to a consumer), in contrast, is a relatively short-term process, but the environmental conditions during this period can deviate considerably from the optimal values. Of course, maintaining optimal conditions during transportation is also possible, but this would make shipping more complicated and expensive. Thus, high storage and transportation potential is an essential prerequisite for a successful and cost-effective inundative biocontrol.

Generally speaking, the successful storage and transportation of an insect in the absence of its natural food depend on two main factors: species-specific starvation resistance (or the ability to survive on factitious food) and environmental conditions. In most aphidophagous ladybirds, both the starvation resistance and the ability to switch to an alternative food are relatively high because in nature aphids represent a very unstable source of food: an aphid colony can rapidly reach a high population density and then quickly disappear [7,8,9]. The most important abiotic environmental factors influencing the success of storage are temperature and humidity. A low temperature decreases their metabolism, thereby increasing resistance to starvation and other adverse factors. On the other hand, too low a temperature—approaching the tolerance limit—becomes an unfavorable factor. High air humidity helps an insect to cope with the desiccation which often accompanies starvation, although too high a humidity can promote the growth of mold or insect pathogens.

The object of our study, the predatory ladybird *Cheilomenes propinqua vicina* Mulsant (=*Cydonia (Cheilomenes) vicina* Mulsant) is widely distributed across Africa and the Middle East [10]. This polyphagous predator was found on various field crops, as well as in vineyards and orchards feeding on various aphids, soft scales, mealybugs, whiteflies, and psyllids [11,12,13,14,15,16,17,18,19,20,21,22,23,24,25,26,27]. In particular, *C. propinqua* was shown to be an important natural enemy of such serious pests as aphids *Myzus persicae* (Sulzer) [22,23], *Aphis gossypii* Glover [11,16,23,24,25,27], *Aphis fabae* Scopoli [21], *Aphis craccivora* Koch [24] and *Lipaphis erysimi* (Kalt) [23], soft scales of the genus *Saissetia* Deplanche [14], mealybugs *Ferrisia virgata* (Cockerell) [12] and *Phenacoccus solenopsis* Tinsley [26], whiteflies *Bemisia tabaci* (Gennadius) and *Aleurodicus dispersus* Russell [13], and citrus psylla *Trioza erytreae* (Del Guercio) [17]. However, we consider *C. propinqua* as a potential mass-reared agent for the biological control of greenhouse pests. In this context, it should be noted that—under the optimal conditions—this relatively small (body length 4–5 mm) and rapidly developing (10–11 days from egg to adult stage) ladybird shows rather high voracity (females consumed 40–80 aphids per day, depending on the prey species; when feeding on *A. gossypii*, larvae consumed a total average of about 500, and females consumed about 2000 individuals) and fecundity (15–30 eggs per day, with a total lifetime fecundity of about 1000 eggs per female) [21,28,29,30]. Such a combination of beneficial properties suggests that *C. propinqua*, as well as some other coccinellids [31], can be an effective agent for biological control. Indeed, greenhouse trials conducted in 2019–2020 in East Africa showed that *C. propinqua* can successfully control *A. fabae* on kalanchoe crops [21].

The use of an insect predator for the biological control of pests of protected crops, as was noted above, involves the development of optimal methods for its mass rearing, storage, packaging, distribution, and release, which in turn requires a basic knowledge of its ecophysiology. The influence of the temperature and prey species on the rate of development, fecundity, mortality, net reproductive rate, and some other biological parameters of *C. propinqua* has been investigated [11,20,28,30]. The storage potential, however, was not studied. The aims of the present study were to estimate the ability of *C. propinqua* females to survive and to retain reproductive potential in the absence of natural food under various hydrothermal and trophic conditions, and thereby to select the optimal methods for their storage and transportation.

## 2. Materials and Methods

The experiments were conducted with a laboratory population of *C. propinqua* originating from 42 adults collected on 10–14 September 2015 in Alexandria (31.200391° N, 29.9155046° E) and reared in the All-Russian Institute of Plant Protection on the wheat aphid (*Schizaphis graminum* Rond.) at a temperature of 24 °C and in a photoperiod of L:D = 16:8 (hereafter, light and dark periods are given in h). Before the study, the ladybirds were reared for several generations in the Laboratory of Experimental Entomology, Zoological Institute RAS, in glass cylinders covered with nylon tissue at a temperature of 25 °C and L:D = 16:8, feeding on nymphs and adults of the green peach aphid *Myzus persicae* (Sulz.), which were reared on *Vicia faba* L. seedlings.

To start the experiment, a cohort of the first instar larvae hatched over 24 h from eggs laid by 10–20 *C. propinqua* females was reared on the green peach aphid at a temperature of 25 °C and L:D = 10:14, i.e., under the short-day conditions that increase the survival of starving females [28]. The emerging adults were kept in groups of 20–50 individuals under the same conditions for 5 more days. Then, randomly selected females were distributed among the experimental treatments.

The study included two experiments. The aim of the first, ‘short-term’ experiment was to imitate short-term storage or transportation to a consumer, whereas the second, ‘long-term’ experiment aimed to estimate the long-term storage potential at the rearing facilities. In both experiments, females were stored in plastic Petri dishes (90 mm × 15 mm) lined with paper (9–10 individuals per dish). The dishes were placed in tightly (but not hermetically) closed plastic containers.

The experimental treatments differed in the following factors.

1. Duration of storage: 3, 6, 9, and 12 days in the short-term experiment; 20, 40, 60, and 80 days in the long-term experiment; and 0 (no storage) in the controls.
2. Temperature: 7, 12, 17, 22, and 27 °C in the short-term experiment; 12, 15, and 17 °C in the long-term experiment (in all of the treatments, the insects were stored in the dark). These ranges of temperatures were used because we presumed that the storage temperature at the rearing facility can be strictly controlled, whereas the transportation conditions can vary considerably. Regarding the long-term experiment, our (unpublished) pilot test demonstrated that at temperatures of 10 °C and lower *C. propinqua* adults are not able to feed, whereas the results of our previous study [28] suggest that feeding on some factitious food is necessary for their long-term storage. On the other hand, another earlier study [30] showed that at 20 °C (in contrast to 15 °C) *C. propinqua* females matured and laid eggs, suggesting that the temperature of 20 °C is too high for long-term storage.
3. Humidity: in both experiments, two levels of air humidity were used, i.e., low (about 50%) and high (80–90%). The low humidity was maintained using a saturated solution of calcium nitrate, and the high humidity was maintained using saturated sucrose solution.
4. Trophic regime: fed vs. starved. Frozen eggs of the Angoumois grain moth *Sitotroga cerealella* Oliv. glued to a piece of hard paper with 30% sugar solution were used as a factitious food. In the long-term experiment, this food was offered to all of the females, whereas in the short-term experiment, it was provided in only half of the treatments (excluding those with a temperature of 7 °C, which—as was noted above, —is too low for feeding). The food was always provided in excess, as needed (e.g., daily at 27 °C and weekly at 12 °C).

After the end of the storage period, all of the surviving females were used for the estimation of their reproductive potential. To this aim, they were individually placed in the same size (90 mm × 15 mm) Petri dishes and kept for 20 days under the optimal conditions (25 °C and L:D = 16:8), feeding on the green peach aphid. The eggs laid during this period were counted daily. In addition, a batch of eggs laid during this period was incubated under the same condition, and the hatching percentage was calculated. At the end of the estimation period, the hind femur length of each female was measured using a stereo microscope with an accuracy of 0.025 mm as an indicator of its size, and then the experiment was finished (females that died during the storage or estimation period were also measured). Thus, the percentage of females that survived to the end of the storage period was calculated for each treatment of each experiment, and for each female the number of eggs laid over 20 days, the percentage of hatched eggs, and the hind femur length were recorded. Then, the final reproductive potential was calculated for each female as the product of fecundity and the percentage of hatched eggs (the reproductive potential of females that did not survive the storage period was taken to be zero).

The short-term experiment included 72 treatments (combinations of 4 storage periods, 5 temperatures, 2 levels of humidity, and 2 trophic regimes for 4 of the 5 temperatures). The long-term experiment included 24 treatments (combinations of 4 storage periods, 3 temperatures, and 2 levels of humidity). In addition, control treatments were carried out, in which the females were not stored. The experiments were performed with 27–30 females per experimental treatment, and 58 control females (a total of 2665 females were used in the present study).

For the statistical analysis of the binary parameter (survival), probit analysis was used; the treatments were compared by the chi-square test. The fecundity and the percentage of hatched eggs were analyzed by GLM and ANOVA. For the final reproductive potential (which was often bimodally distributed), a non-parametric Kruskal–Wallis test was used. All of the calculations were conducted using SYSTAT 10.2 (http://systat.software.informer.com/10.2 (accessed on 1 July 2022)).

## 3. Results

### 3.1. Survival

**Figure 1:**
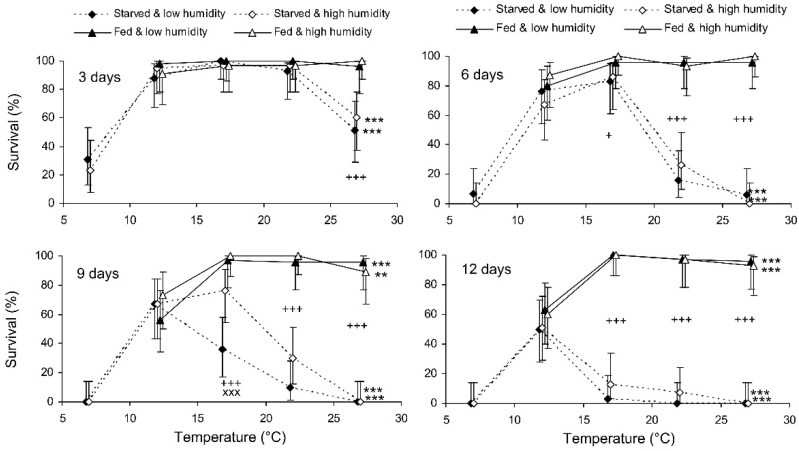
Survival of *Cheilomenes propinqua* females in relation to the temperature, humidity, trophic regime, and duration of storage (the results of the short-term experiment). Percentages with 95% confidence intervals are shown. The duration of storage is indicated in the graphs. Asterisks at the right end of the line indicate the significant influence of temperature: **—*p* < 0.01 or ***—*p* < 0.001 by the chi-square test. Plus symbols indicate a significant influence of the trophic regime at the corresponding temperature: +—*p* < 0.05, +++—*p* < 0.001, according to the Mantel–Haenszel chi-square test (humidity was used as a strata variable). X-crosses indicate the significant influence of humidity at the corresponding temperature, ×××—*p* < 0.001, according to the Mantel–Haenszel chi-square test (the trophic regime was used as a strata variable).

**Figure 2:**
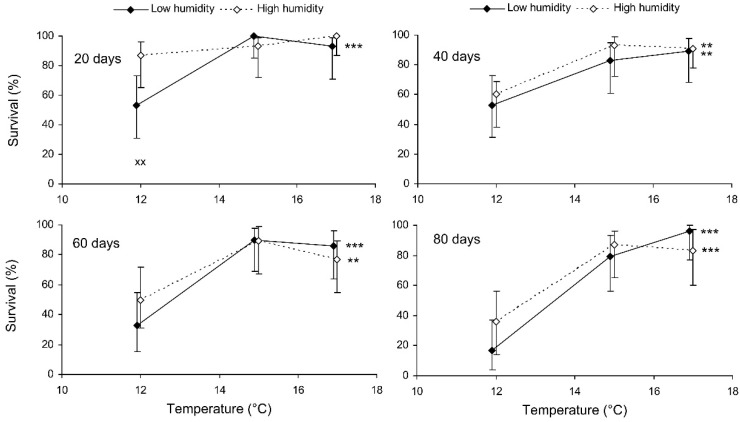
Survival of fed *Cheilomenes propinqua* females in relation to the temperature, humidity, and duration of storage (the results of the long-term experiment). Percentages with 95% confidence intervals are shown. The duration of storage is indicated in the graphs. Asterisks at the right end of the line indicate a significant influence of temperature—**, *p* < 0.01, ***, *p* < 0.001—according to the chi-square test. X-crosses indicate the significant influence of humidity at the corresponding temperature, ××, *p* < 0.01, according to the Mantel–Haenszel chi-square test (the trophic regime was used as a strata variable).

The binary probit analysis of the results of the short-term experiment showed that, as expected, the survival of *C. propinqua* females decreased with the storage period, and fed individuals survived better than starved ones (Table 1). In addition, survival decreased with the temperature and increased with the humidity (although the last effect was marginally not significant). In Figure 1, it can be seen that the survival percentage of starved females decreased at extreme temperatures of 7 and 27 °C. Fed females did not show any decrease in survival at 27 °C, but at 12 °C their survival decreased after 9 days of storage (reminder: the storage potential of fed females was not tested at 7 °C because feeding is not possible at this temperature). In total, the highest survival of starved females was observed at 12 °C, whereas fed females showed equally high survival at a wide range of temperatures from 17 to 27 °C. The impact of the trophic factor increased with the temperature: at 27 °C, the difference in survival between starved and fed females was already significant after 3 days of storage, whereas at 12 °C it was not significant even after 12 days. The survival of starved females after 6 and more days of storage tended to increase with the humidity, but in most cases this effect was not statistically significant. The long-term experiment gave similar results (Table 1, Figure 2). The survival of fed females at 15–17 °C was independent of the humidity, and was markedly higher than that at 12 °C. Under the optimal conditions, about 80% of the females survived for at least 80 days.

### 3.2. Fecundity

**Figure 3:**
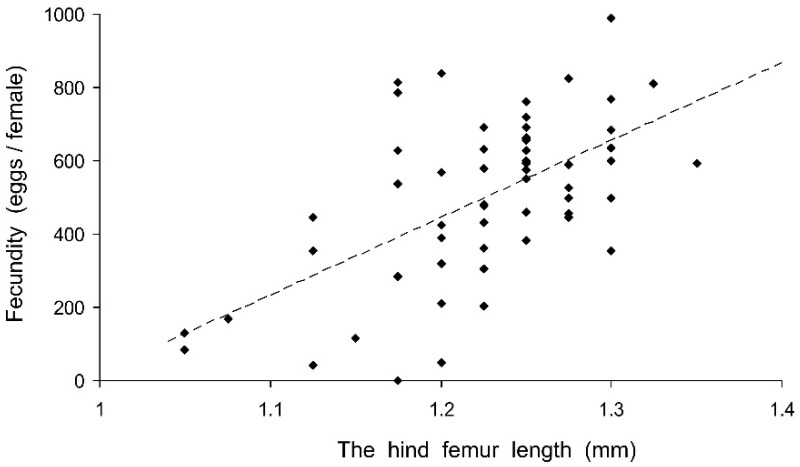
Fecundity of *Cheilomenes propinqua* females in relation to their size, as estimated by the hind femur length. Each symbol corresponds to one control female. The dashed line shows the linear regression Y = 2079 X − 2046 (r = 0.584, *n* = 58, *p* < 0.001).

**Figure 4:**
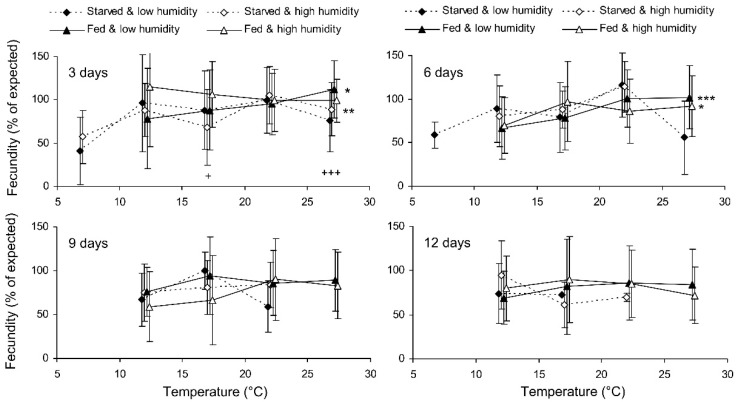
Fecundity of *Cheilomenes propinqua* females in relation to the temperature, humidity, trophic regime, and duration of storage (the results of the short-term experiment). The means and SD of the percentage of the expected fecundity calculated based on the female size are shown. The duration of storage is indicated in the graphs. Asterisks at the right end of the line indicate a significant influence of temperature—*—*p* < 0.05, **—*p* < 0.01, ***—*p* < 0.001—according to a one-way ANOVA test. Plus symbols indicate the significant influence of the trophic regime at the corresponding temperature, +—*p* < 0.05, +++—*p* < 0.001, according to a two-way ANOVA test (humidity was the second factor).

**Figure 5:**
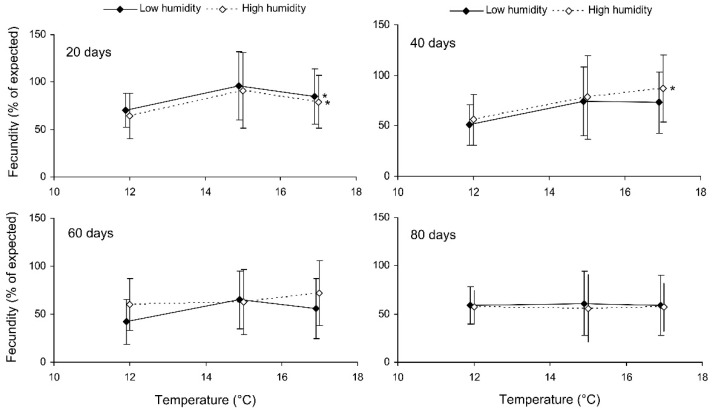
Fecundity of fed *Cheilomenes propinqua* females in relation to the temperature, humidity, and duration of storage (the results of the long-term experiment). The means and SD of the percentage of the expected fecundity calculated based on the females’ size are shown. The duration of storage is indicated in the graphs. Asterisks at the right end of the line indicate a significant influence of temperature, *—*p* < 0.05, according to a one-way ANOVA test.

Preliminary analysis of the data for the controls showed that their fecundity positively correlated with size, which was highly variable both in the control and experimental females. Therefore, the data were transformed to exclude the ‘size factor’. First, the expected fecundity was calculated for each female based on the regression equation for the controls (Figure 3). Second, the observed fecundity was expressed as a percentage of the expected fecundity with the formula Ft = 100 (Fo/Fe), where Ft is the transformed fecundity, Fo is the observed fecundity, and Fe is the expected fecundity. Then, this percentage was used for further statistical treatment. The GLM analysis of the total results of the short-term experiment showed that the fecundity of the surviving females increased with the temperature and decreased with the duration of storage, whereas the impacts of the humidity and trophic regime were not significant (Table 2). However, the analysis of some particular datasets revealed the significant difference between fed and starved individuals (Figure 4). The long-term experiment yielded very similar results (Table 2, Figure 5). In general, the influence of environmental factors on the fecundity of *C. propinqua* females was weaker than that on survival (compare Figure 1 and Figure 4, Figure 2 and Figure 5).

### 3.3. Egg Hatching

**Figure 6:**
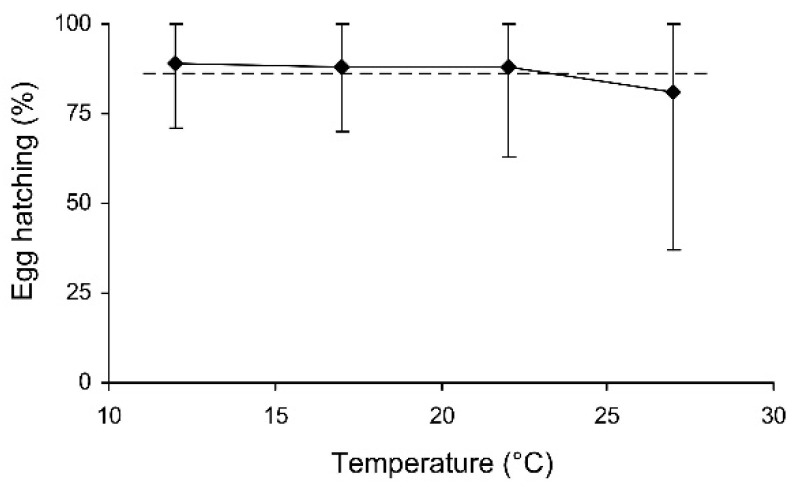
The percentage of hatched eggs of *Cheilomenes propinqua* females in relation to the temperature (the results of the short-term experiment). Medians and quartiles are shown. The dashed line shows hatching in the controls.

**Figure 7:**
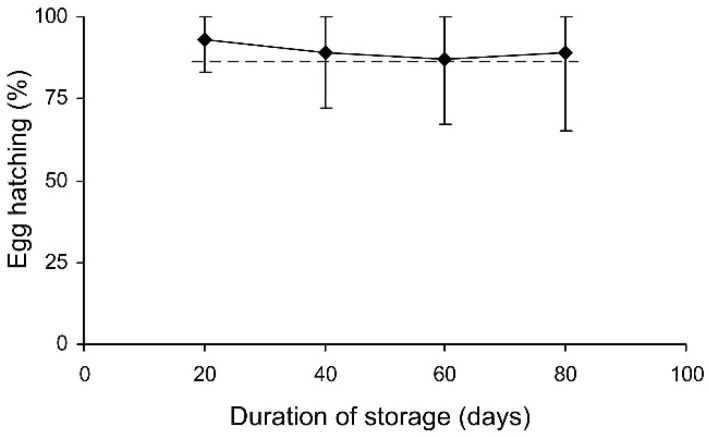
The percentage of hatched eggs of *Cheilomenes propinqua* females in relation to the duration of storage (the results of the long-term experiment). Medians and quartiles are shown. The dashed line shows hatching in the controls.

In the short-term experiment, the percentage of hatched eggs was somewhat lower in females which were stored at 27 °C (Table 3, Figure 6). In the long-term experiment, the thermal effect was not significant (evidently because of the limited range of temperatures used), but egg hatching slightly decreased with the duration of storage (Table 3, Figure 7). However, in both experiments the percentage of hatching of the eggs laid by the experimental females was very close to that of the controls (Figure 6 and Figure 7).

### 3.4. Final Reproductive Potential

**Figure 8:**
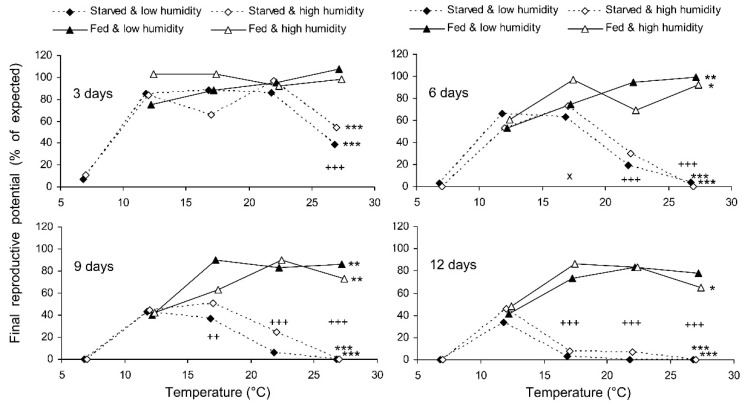
Final reproductive potential of *Cheilomenes propinqua* females in relation to the temperature, humidity, trophic regime, and duration of storage (the results of the short-term experiment). Asterisks at the right end of the line indicate a significant influence of temperature: *—*p* < 0.05, **—*p* < 0.01, ***—*p* < 0.001, according to the Kruskal–Wallis test. Plus symbols indicate a significant influence of the trophic regime at the corresponding temperature: ++—*p* < 0.01, +++—*p* < 0.001, according to the Kruskal–Wallis test. X-crosses indicate the significant influence of humidity at the corresponding temperature: ×—*p* < 0.05 according to the Kruskal–Wallis test.

**Figure 9:**
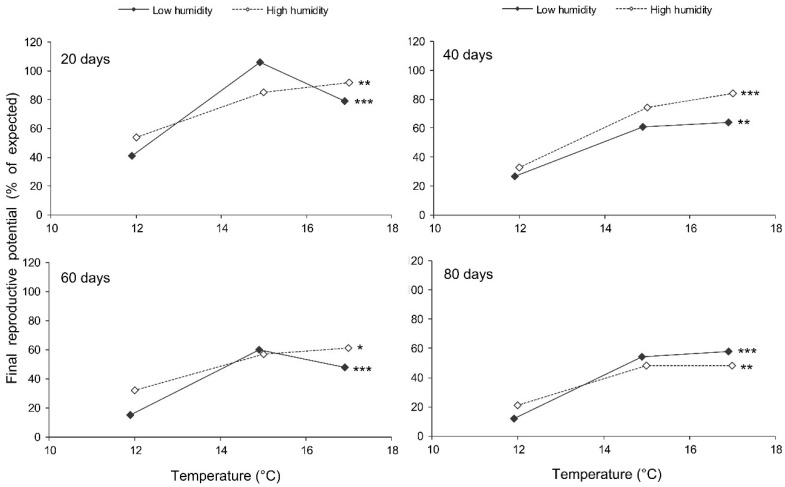
Final reproductive potential of fed *Cheilomenes propinqua* females in relation to the temperature, humidity, and duration of storage (the results of the long-term experiment). Asterisks at the right end of the line indicate the significant influence of temperature: *—*p* < 0.05, **—*p* < 0.01, ***—*p* < 0.001, according to the Kruskal–Wallis test.

The final reproductive potential, an estimation of the mean number of progeny per female (including both individuals that survived the storage and those that did not) was expressed as the percentage of the expected value calculated with the same formula that was used for fecundity. This indicator represents a product of the three above parameters (survival, fecundity, and hatching); therefore, it shows the same dependencies on the same factors. In the short-term experiment, the fed females retained high reproductive potential when stored at a wide range of temperatures from 17 to 27 °C, both at low (about 50%) and at high (80–90%) air humidities. Under these conditions, their reproductive potential even after 12 days of storage constituted about 80% of that in controls (Figure 8). The starved females retained about 80% of the initial reproductive potential for only 3 days of storage at 12–22 °C. Then, their reproductive potential gradually decreased, being the highest in females stored at 12 °C (about 40% of that in controls after 12 days of storage). In the long-term experiment, the best results were obtained at 15 and 17 °C: the reproductive potential was about 80% and 50% of that in controls after 20 and 80 days of storage, respectively (Figure 9).

## 4. Discussion

The storage potential of predatory ladybirds used for the biological control of insect pests varies greatly among species. For example, Semyanov [32] reported the mortality after 3 months’ storage under the standard conditions (12 °C, feeding on sucrose solution) of *Cheilomenes (Menochilus) sexmaculata* was 80%, that of *Leis biplagiata* was 50%, that of *Harmonia sedecimnotata* was 30%, and that of *Leis dimidiata* was 4% (in the last species, the mortality was 13–14% after 6 months of storage), and suggested that the storage potential is largely determined by the adult size. Other authors using other methods of storage also reported very different species-specific storage potentials [6]. The longest shelf life, as expected, was observed in Coccinellidae species of the temperate climate entering the long winter diapause in nature. For example, several authors demonstrated the high survival and relatively high fecundity of *Harmonia axyridis* after very long (up to eight months) storage [33,34,35,36,37].

The optimal storage conditions also correlate with the natural habitats of a ladybird. The above-mentioned *H. axyridis* (its geographic range includes Southern Siberia), as well as some other species of the temperate zone, show maximal storage potential at near-zero temperatures [33,34,37,38,39], whereas the subtropical and tropical ladybirds *Scymnus coccivora* and *C. propinqua* should be stored at 15–20 °C [40].

In practice, based on the results of the present study, we conclude that:

- In rearing facilities, *C. propinqua* females can be successfully stored for future use in mass rearing or in the biological control of pests. Under the optimal conditions (temperatures of 15–17 °C, air humidities of 50–90%, feeding on the frozen eggs of the grain moth) females can be stored for 20 days with a rather low (about 20%) and for up to 80 days with an acceptable (not more than 50%) loss of the reproductive potential. Moreover, about 80% of beetles not only survive 80 days storage but retain relatively high viability for another 20 days. Although the fecundity of these females significantly decreases, it is still enough to eliminate an initial aphid colony which usually represents a progeny of a single foundress.
- Short-term (up to 3 days) storage or transportation (shipment) can occur at a much wider range of temperatures: from 12 to 27 °C and from 12 to 22 °C for fed and starved females, respectively. Moreover, the mortality of fed females stored for up to 12 days at temperatures of 17–27 °C was still not higher than 10%, although in the absence of food they could be stored at 12–17 °C for not more than 6 days.
- In greenhouses, *C. propinqua* can be successfully used for the biological control of aphids by preventing colonization (a “standing army” approach [4,5,41,42,43]). Indeed, considering the 20-day viability of adults after long storage, their biweekly releases would be enough to protect the plants without even relying on the predators’ progeny. It should, however, be noted that the application of this method would require the regular supplementation of factitious food, as is also recommended by many biocontrol practitioners [4,5,41,42,43,44,45].
- Storage for more than 10 days at temperatures of 7 °C and lower results in the 100% mortality of both starved and fed females of *C. propinqua.* Hence, beetles of the studied population which escape from greenhouses or rearing facilities would not survive even the mild winter of the south temperate climate zone.

## Figures and Tables

**Table 1:** Influence of the trophic regime, temperature, humidity, and duration of storage on the survival of *Cheilomenes propinqua* females (the results of the binary probit analysis: the regression coefficient ± SE, *t*-ratio, and significance of influence).

Factor	The Short-Term Experiment (*n* = 1899)	The Long-Term Experiment (*n* = 708)
Trophic regime	1.705 ± 0.079, *t* = 21.5, *p* < 0.001	– ^1^
Temperature	−0.048 ± 0.006, *t* = −7.6, *p* < 0.001	0.300 ± 0.028, *t* = 10.6, *p* < 0.001
Humidity	0.004 ± 0.002, *t* = 1.8, *p* = 0.066	0.006 ± 0.003, *t* = 1.9, *p* = 0.053
Duration of storage	−0.173 ± 0.012, *t* = −14.7, *p* < 0.001	−0.014 ± 0.003, *t* = −5.3, *p* < 0.001

^1^ No data.

**Table 2:** Influence of the trophic regime, temperature, humidity, and duration of storage on the fecundity of *Cheilomenes propinqua* females (the results of the GLM analysis: the regression coefficient ± SE, *t*-ratio, and significance of influence).

Factor	The Short-Term Experiment (*n* = 1285)	The Long-Term Experiment (*n* = 523)
Trophic regime	2.944 ± 2.572, *t* = 1.1, *p* = 0.253	– ^1^
Temperature	0.845 ± 0.213, *t* = 4.0, *p* < 0.001	3.009 ± 0.742, *t* = 4.1, *p* < 0.001
Humidity	0.006 ± 0.065, *t* = 0.1, *p* = 0.920	0.045 ± 0.079, *t* = 0.6, *p* = 0.567
Duration of storage	−1.604 ± 0.349, *t* = −4.6, *p* < 0.001	−0.439 ± 0.062, *t* = −7.1, *p* < 0.001

^1^ No data.

**Table 3:** Influence of the trophic regime, temperature, humidity, and duration of storage on the percentage of hatched eggs in *Cheilomenes propinqua* (the results of the GLM analysis: the regression coefficient ± SE, *t*-ratio, and significance of influence).

Factor	The Short-Term Experiment (*n* = 1216)	The Long-Term Experiment (*n* = 495)
Trophic regime	−2.525 ± 2.187, *t* = 1.1, *p* = 0.249	– ^1^
Temperature	−0.655 ± 0.181, *t* = −3.6, *p* < 0.001	0.447 ± 0.730, *t* = 0.6, *p* = 0.541
Humidity	−0.076 ± 0.055, *t* = −1.4, *p* = 0.167	0.035 ± 0.078, *t* = 0.5, *p* = 0.652
Duration of storage	−0.346 ± 0.297, *t* = −1.2, *p* = 0.244	−0.151 ± 0.062, *t* = −2.4, *p* = 0.015

^1^ No data.

## Data Availability

Data supporting the reported results can be obtained upon request from the corresponding author (S.Y.R.).
